# Advancing global health through cardiovascular research, mentorship, and capacity building: in memoriam, professor Bongani Mayosi (1967–2018)

**DOI:** 10.1186/s40814-018-0348-7

**Published:** 2018-10-03

**Authors:** Jean B. Nachega, Mpiko Ntsekhe, Jimmy Volmink, Lehana Thabane

**Affiliations:** 1 McMaster University, Hamilton, ON Canada; 2 Stellenbosch University, Stellenbosch, South Africa; 3 University of Cape Town, Cape Town, South Africa; 4 Stellenbosch University, Stellenbosch, South Africa


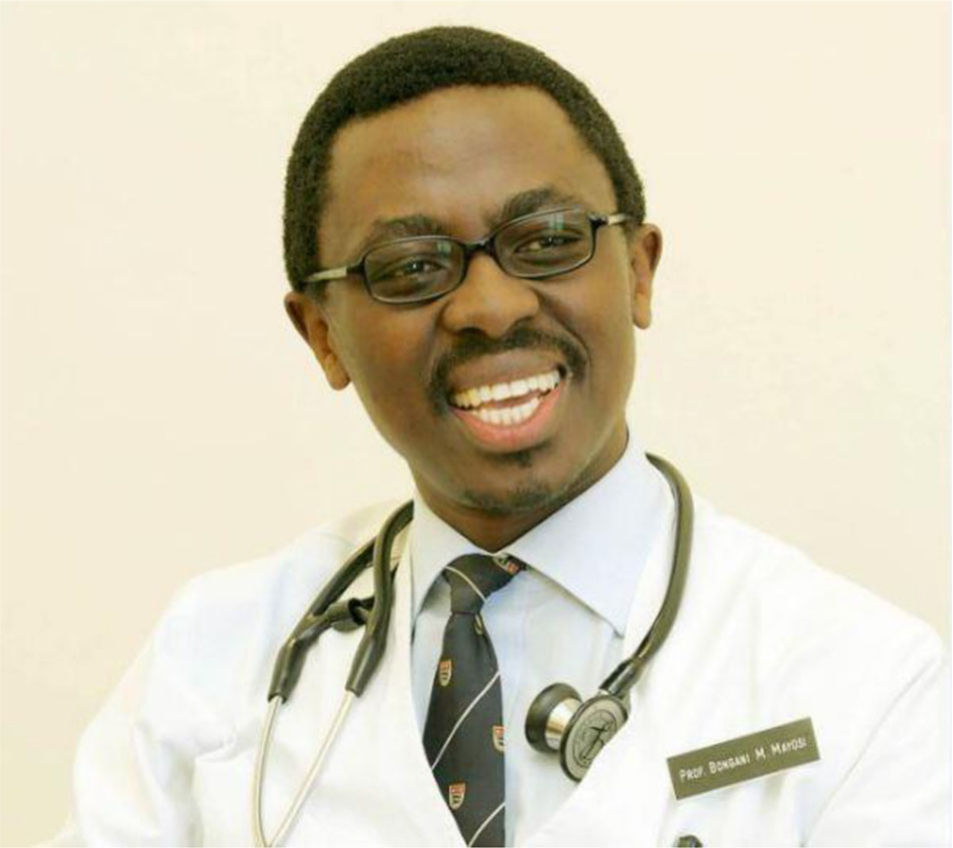


We are deeply saddened by the passing of Professor Bongani Mayosi. Bongani was one of the inaugural board members of *Pilot and Feasibility Studies*. He contributed greatly to the design and conduct of pilot and feasibility studies in cardiovascular research. Before his untimely death on Friday, July 27, 2018, he rose rapidly through the ranks to become a top cardiologist and one of the premier medical researchers in South Africa, Africa and the World

Born in Mthatha, Eastern Cape Province on January 28, 1967, Bongani Mawethu Mayosi followed in his father’s footsteps to become a doctor. He trained at the now Nelson R. Mandela School of Medicine at University of KwaZulu-Natal, where he received his M.B., Ch.B. (*Cum Laude*) in 1989 and also met his wife, Professor and Head of Dermatology, Nonhlanhla Khumalo, in their first week of medical school. In 1990, the pair made their way to Port Elizabeth to work at the Livingstone Hospital as interns, before moving to Cape Town to establish long-term careers. After completing his specialist training in internal medicine and cardiology at the University of Cape Town (UCT), Professor Mayosi moved to Oxford University, UK, on a prestigious Nuffield Medical Fellowship where he completed a D.Phil. in cardiovascular genetics at the Wellcome Trust Centre for Human Genetics.

Upon returning to Cape Town in 2001‚ Professor Mayosi assumed research‚ teaching, and clinical responsibilities in Internal Medicine and Cardiology at UCT and Groote Schuur Hospital, coincidentally the place in which the world’s first human heart transplant took place during the year of his birth. Five years later‚ at the age of 38‚ he became the first black faculty member to be made Professor and Head of the Department of Medicine.

## Contributions to *Pilot and Feasibility Studies*

Overall, Bongani believed strongly in the value of assessing feasibility to ensure the successful conduct of main trials—something that he applied in the design of his IMPI (investigation of the management of pericarditis) trial to determine the effects of prednisolone and *Mycobacterium indicus pranii* (Mw) immunotherapy on the composite outcome of death, constriction, or cardiac tamponade requiring pericardial drainage in patients with TB pericardial effusion [1]. He was among the first international colleagues to answer the call to serve on the editorial board of the journal when it was launched in 2015. Bongani truly believed in the mission of the journal—to provide dedicated space for both the reporting of feasibility and pilot studies, as well as discussion of methodological issues around the planning of future large-scale definitive studies—as a way to advance health not only in South Africa but around the world. Despite his busy schedule as Dean of the Faculty of Health Sciences at UCT, Bongani still took time to review manuscripts for Pilot and Feasibility Studies, which he did as recently as in June 2018. We owe the success of the journal, in part, to the dedication of scientists like Bongani.

## Contributions to mentoring and research capacity building in Africa

Perhaps one of Bongani’s most remarkable legacies is his dedication to mentoring junior clinician scientists and building research capacity in Africa [2]. In 2007, citing difficulties faced during his own research training, this visionary played a leading role in the launch of the South African National Health Scholars Programme (1000 Ph.D. scholarships during a 10-year initiative) [3]. With an eye on the future of building research capacity in hopes of improved clinical outcomes, he maintained a thriving translational research lab. Google “bongani mayosi and mentoring” you will get over 4000 hits, most of which are commentaries, press or media news, tributes, or sentiments expressed by the many people lucky to have known him or have been mentored by him. He was a natural born teacher who made a deliberate decision to share his knowledge with others.

Bongani was a true genius: his calmness was soothing in the face of chaos; his humility was inspirational; his scientific intuition and insights were brilliant; and his professional and personal instincts, truly amazing. These are the qualities that made him a magnet for young researchers who came from all over the African continent—Nigeria, Botswana, Lesotho, Swaziland, Malawi, Zambia, Kenya, Uganda, and Zimbabwe—to have the privilege of being mentored by one of its top cardiologists. One of us (MN), who is now head of the Division of Cardiology at UCT, is one of the grateful beneficiaries of his mentorship.

## Contributions to cardiovascular science

While Professor Christian Barnard put South Africa on the map by performing the world’s first heart transplant on December 3, 1967 [4], Professor Bongani Mayosi made his country famous for its global leadership in cardiovascular clinical research. A core part of his research mission was to prioritize cardiovascular diseases of poverty which (a) disproportionately affected Africans and (b) had been neglected by the global research agenda. In 2008, he led the multinational IMPI trial which investigated the use of steroids in treating tuberculosis pericarditis [1]. IMPI found that among patients with tuberculous pericarditis, steroids (i) do not reduce the incidence of the combined outcome of death, cardiac tamponade, or constriction; (ii) increase the incidence of HIV-associated cancer; (iii) reduce the incidence of constrictive pericarditis and hospitalization regardless of HIV status; and (iv) Mw is ineffective but increases the incidence of HIV-associated cancer. The findings of IMPI have set a new standard for clinical practice in the world. He then led the first phase of a large-scale‚ multinational study of rheumatic heart disease, the leading cause of heart disease among school children, in Africa [5, 6]. Venturing into yet another clinical challenge, Professor Mayosi was perplexed by how highly athletic persons could experience life-threatening abnormal rhythms of the heart during routine exercise. His team, joined by others across the globe, proposed CDH2 mutations as novel genetic causes of arrhythmogenic right ventricular cardiomyopathy (ARVC). CDH2 encodes cadherin 2 (also known as N-cadherin), a protein that plays a vital role in cell adhesion, making it a biologically plausible candidate gene in ARVC pathogenesis. Little did we know that this groundbreaking discovery was to be his swansong [7].

Having co-authored over 330 peer-reviewed publications, Bongani’s contributions to science are truly remarkable. He received many honors and awards during his stellar career. Those that he particularly cherished include: South Africa’s highest presidential award, the Order of Mapungubwe in Silver (*in recognition of excellent contributions to medical science*) in 2009; the National Science and Technology Foundation—BHP Billiton Award (*To an individual for outstanding contribution to Science Engineering Technology and Innovation Through Management and related activities over the previous 5–10 years or less*) in 2012; the National Research Foundation (NRF) Science Team Award in 2017. This award recognizes that it is often teams working collaboratively that produce the types of research that profoundly benefit society. This was the case for Professor Mayosi’s discovery of the gene responsible for ARVC, which came after 20 years of research and international collaboration across four countries on three continents; the Honorary Fellowship of Wolfson College, University of Oxford (*to individuals whom they particularly value and admire for their outstanding distinction in their field*); and in 2016, an NRF “A” Rating (*for researchers who are unequivocally recognized by their peers as leading international scholars in their field for the high quality and impact of their recent research outputs*). Finally, in 2017, he became the only African admitted that year to the US Academy of Medicine.

Bognani was a true global citizen, who always carried the torch for South Africa and Africa. His role as a member of editorial boards of several national and international journals, included that of Associate Editor for Africa of *Circulation*. He was also the President of the College of Physicians of South Africa, Chairman of the South African National Health Research Committee, President of the Pan-African Society of Cardiology (PASCAR), and Chairman of the Advisory Committee of Health Research and Development of the World Health Organization—Africa Region. In 2007, he convened the historic first “All Africa, All Heart” Conference of PASCAR that was held in Nairobi, Kenya from 13 to 16 May 2007—followed by similar meetings in Abuja, Nigeria (2009); Kampala, Uganda (2011); Dakar, Senegal (2013); and Balaclava, Mauritius (2015)—that revitalized the cardiovascular medicine community on the African continent.

Professor Mayosi was the South African lead investigator on many international studies that have contributed to a better understanding of cardiovascular disease prevention, treatment, and management globally. These include: IMPI trial [1] and the PURE [8], PASCAR [9], MANAGE [10], and REMEDY [5, 6] studies. He played a leading role in establishing the Global Rheumatic Heart Disease Registry (the REMEDY study) to provide information on the clinical characteristics, treatment, outcomes, and barriers to care of rheumatic heart disease. Being the first and largest multi-center study of rheumatic heart disease, REMEDY showed that this disease of the young is associated with a heavy burden of complications and identified major gaps in the use of proven interventions such as penicillin for secondary prophylaxis and oral anticoagulants for those at risk for stroke [5, 6]. Professor Mayosi’s research is documented in many leading, high-impact medical journals such as NEJM, JAMA, Lancet, BMJ, Circulation, European Heart Journal, the American Journal of Human Genetics, Heart, American Heart Journal, and PloS ONE, to mention only a few. His health research collaborations span the entire globe and include researchers from 39 countries. In all these efforts, Bongani always extended opportunities to other African researchers. For example, his IMPI trial included seven African countries (South Africa, Zimbabwe, Mozambique, Malawi, Sierra Leone, Nigeria, and Kenya).

## Personal reflections

Bongani, a true family man, is survived by his wife, Professor Nonhlanhla Khumalo, Head of the Dermatology Department at UCT—who is a renowned researcher and educator in her own right, and three daughters, Nosipho‚ S’vuyile Mayosi-Manana, who followed in her parents and grandfather’s footsteps to become a doctor, and Camagu. In January this year, Nonhlanhla organized a surprise party for him in the majestic mountains of Stellenbosch in Cape Town to celebrate his 51st birthday with friends and family. All those who attended effused about his contributions to their own personal and career growth and development, as well as his impact on medicine in South Africa and beyond as a researcher, educator, and administrator. They talked about his human qualities which were so attractive to the many who aspired to be like him: his work ethic, kindness, generosity, mentorship style, and his infectious smile! These are some of Bongani’s priceless attributes which we will miss, but that will long be cherished in our hearts.

Sadly, a man who touched and inspired so many personally struggled with the isolation and bleakness of a mental illness. After his death, his family released a statement including the following: “In the last two years he has battled with depression and on that day took the desperate decision to end his life” [11].

On the heels of suicides of several other prominent, accomplished, renowned personalities globally, Professor Mayosi’s death confirms that mental illness does not play favorites. No one is immune, regardless of age, race, gender, socio-economic status, or profession. We know that depression and suicide rates among health professionals are even higher than those in the general population [12, 13]. In Africa, discussion about mental health remains taboo because of stigma. The openness of Professor Mayosi’s family in disclosing the cause of his death was commendable and offers a start. Suicide leaves survivors with so many questions. There is a need for ongoing reflection on the death of Professor Mayosi, in order to learn lessons about how such tragic events can be prevented, detected, and managed better within the academic environment [14]. We have a collective responsibility as individuals working within an often fraught health care system to tackle these stigmatizing issues head-on, implement evidence-based prevention strategies (viz. education, confidential screening, and early intervention), and increase awareness of factors that offer protection from suicidal behavior and that promote wellness and recovery. This will require clinical and academic leadership at the highest level to break the culture of silence and support action.

Professor Mayosi was one of a kind; his untimely passing is a tragedy of immense proportions. We can celebrate his life and honor his legacy by continuing with those academic, research, and training programs he was so passionate about. Rest in peace, Bongani: our brother, friend, colleague, and life mentor.
